# 天然中药单体及衍生物调控非小细胞肺癌自噬的研究新进展

**DOI:** 10.3779/j.issn.1009-3419.2017.03.10

**Published:** 2017-03-20

**Authors:** 美亦 相, 瑞蕾 李, 志伟 张, 鑫 宋

**Affiliations:** 650500 昆明，云南省昆明医科大学第三附属医院，云南省肿瘤医院生物治疗中心 Department of Cancer Biotherapy Center, the Third Affiliated Hospital of Kunming Medical University, Kunming 650500, China

**Keywords:** 肺肿瘤, 中药提取物, 自噬, 耐药, Lung neoplasms, Chinese herbal or plant extracts, Autophagy, Drug resistance

## Abstract

非小细胞肺癌（non-small cell lung cancer, NSCLC）发病率、病死率高，亟待研发高抗癌活性、低毒副作用的新型疗法。自噬既可诱发NSCLC癌细胞死亡又可介导死亡逃避，多样化的角色使之成为抗癌的新机制。天然中药众多活性单体及其衍生物具备抗NSCLC的活性，甚至可影响其获得性耐药，而对自噬的调控多被证实为其潜在的作用机制。针对多种天然药物来源的萜类化合物、生物碱、膳食多酚、皂苷类化合物及其它活性成分的研究证实，不同单体成分既可影响NSCLC保护性自噬水平，亦可调控其死亡性自噬活性，相应的影响NSCLC的生长及对某些药物的反应性。因而，本文就天然中药成分及其衍生物通过双向调节自噬活性参与NSCLC治疗的最新进展做一综述。

## Abstract (en)

The high morbidity and mortality of non-small cell lung cancer (NSCLC) did influence the quality of life of tumor patients world-wide. There is an urgent need to develop new therapies that have high anti-tumor activity and low toxicity side effects. It is widely accepted that autophagy can play diverse roles in carcinogenesis, such as induces pro-death of lung cancer cells or helps the escape from cell death, making it become a proper anticancer target. It's believed that various monomers of Chinese traditional medicine closely correlates to anti-NSCLC activities, and that even could affect the acquired multiple drug resistance (MDR). Furthermore, autophagy might be the underling mechanisms which could play a role as the candidate targets of natural active compounds. Recent studies of terpenoids, alkaloid, dietary polyphenols, saponins and other active ingredients that extracted from a large variety of herbs suggest that different monomer compounds could either regulate the activity of pro-death autophagy or influence the level of protective autophagy of NSCLC cells, thus changing their drug sensitivity and cell viability. This paper aims to give a systemic description of the latest advances about natural compounds and their derivatives that involved in tumorigenesis of NSCLC via inducing the autophagy.

原发性支气管肺癌包括非小细胞肺癌（non-small cell lung cancer, NSCLC）和小细胞肺癌，NSCLC占肺癌总体的85%，发病率和病死率高居所有肿瘤首位。化疗药物严重的毒副反应、靶向治疗药物的高耐药率以及肿瘤免疫耐受微环境给肺癌的治疗带来了诸多挑战，亟待探索新型的抗癌疗法^[1]^。2016年诺贝尔生理学或医学奖关注于自噬的内在作用机理，进一步为理解自噬在癌症中发挥的角色提供了理论依据^[2]^。并且新近的研究表明，作为天然药物发挥抗癌作用的重要机制之一，自噬参与到NSCLC发生发展的病理进程中。

天然药物较常规治疗药物具有更丰富的结构和生物活性，已逐渐成为抗癌药物研发的一个巨大资源库^[3]^。中医经历了几千年的文化沉淀，对祖国天然产物药用价值的研究已达到一定高度。近年来，随着对中药抗癌活性认识的不断深入，中药疗法在NSCLC综合治疗中的临床疗效逐渐得到认可，尤其是通过调节自噬影响NSCLC癌细胞的活性及药物敏感性，逐渐成为当前NSCLC用药的研究热点。但是，目前针对不同天然药物调控NSCLC自噬活性的研究尚待完善，其发挥作用的潜在机制远未阐明。因而，明确其自噬相关作用机理对新型抗NSCLC药物的研发意义重大。本文通过总结自噬介导的天然药物的抗癌作用机理，对众多中药单体及其衍生物治疗NSCLC的内在作用机制进行系统综述。

## 1 自噬与癌症的发生

众所周知，目前存在于哺乳动物体内的自噬主要包括巨自噬、微自噬和分子伴侣介导的自噬。在此，我们探讨的自噬主要是指巨自噬，部分情况下又称为Ⅱ型程序性细胞死亡。作为一种进化上高度保守的胞内物质代谢过程，自噬的发生起始于吞噬泡将胞内受损或衰老的细胞器及各类生物大分子包裹形成自噬小体，后与溶酶体融合形成自噬溶酶体，利用酸性水解酶回收氨基酸、脂肪酸和核苷酸等物质，在胞内环境稳态中发挥了关键作用^[4]^。

自噬参与到机体的多系统疾病，且它在NSCLC中发挥的生物学功能尤为复杂。部分观点认为当肿瘤细胞遭遇营养剥夺、抗癌药等环境压力时，升高的自噬活性通过降解掉结构或功能异常的组分和细胞器，协助癌细胞逃避死亡。并且在一定程度上通过维持基因组的稳定性促进癌细胞的生存^[5, 6]^；也有学者指出，诱发的Ⅱ型程序性细胞死亡或自噬功能失调也可抑制癌细胞的活性，或者诱导凋亡发挥间接抑癌的作用，甚至激活自噬可增强肿瘤的治疗效果^[7, 8]^。因而，既可导致NSCLC细胞死亡又可介导死亡逃避的自噬在肿瘤细胞命运决定中以何种作用示人值得探讨。

## 2 多种传统中药单体及其衍生物调控NSCLC的自噬

中药中众多活性单体及其衍生物或类似物克服了传统中药材生物利用度低的限制，对改善NSCLC患者的临床症状及预防复发意义重大。并且癌细胞中的自噬现象与中医的理论也有着异曲同工之处，存在众多从中医角度对自噬的分析。目前用于中药单体与NSCLC治疗临床试验的自噬抑制剂除药品监督管理局（Food and Drug Administration, FDA）批准的氯喹（chloroquine, CQ）外，还包括羟基氯喹（hydroxychloroquine, HCQ）及3-甲基腺嘌呤（3-methyladenine, 3-MA）。其中，氯喹通过影响溶酶体的酸化，阻止自噬体和溶酶体融合抑制癌细胞的自噬^[9]^。通过上述调控剂的参与，抗癌天然药物成分对自噬的调节引发癌细胞死亡或增强癌细胞存活的双重能力在研究中逐渐浮现（图 1），促进了对NSCLC中药治疗的理解。

**1:**
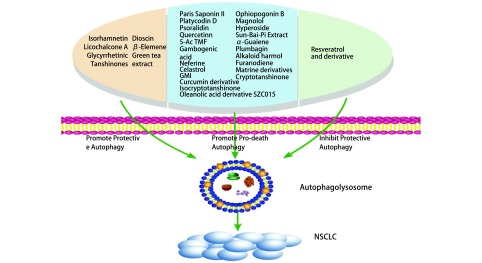
中药单体及其衍生物通过自噬调控NSCLC的细胞活性 Traditional chinese medicine monomers and their derivatives modulate non-small cell lung cancer cell viability through autophagy

### 2.1 多种天然中药活性成分诱发死亡性自噬（pro-death autophagy）抑制NSCLC生长

许多中药来源的活性单体可通过诱发死亡性自噬抑制NSCLC，应用自噬抑制剂逆转自噬多同时抑制凋亡。目前的研究主要集中在丹参酮等天然萜类化合物、甲基莲心碱类生物碱、重楼皂苷等皂苷类物质、姜黄素等膳食多酚、新藤黄酸以及其它活性产物上。

愈创木醇、呋喃二烯、丹参酮以及齐墩果酸等天然萜类化合物改变NSCLC的自噬活性发挥抑癌作用。其中，愈创木醇（-）-Guaiol为愈创木烷型天然产物的重要组分^[10]^，此倍半萜类天然产物作为DNA损伤的诱导剂，不仅阻滞A549细胞于S期并升高早期、晚期和总凋亡的比例，还可诱导自噬减少同源重组修复中关键因子RAD51的表达，促进DNA双链受损的积累协同抑癌。甚至可协助降低NSCLC吉非替尼获得性耐药的发生机率^[11]^。另外，从具有抗癌活性的温郁金的根茎中提取获得的中药单体——呋喃二烯，可通过增强自噬标记分子LC3-Ⅱ的表达诱导自噬，并呈剂量依赖性地阻滞G_1_期进展，抑制肺癌细胞增殖并诱导凋亡^[12]^。众所周知，丹参酮为丹参中主要的脂溶性菲醌化合物^[13]^，其许多组分对NSCLC自噬活性的调控值得关注。例如隐丹参醌可呈浓度和时间依赖性地诱导胞内活性氧（reactive oxygen species, ROS）的形成，激活JNK信号通路^[14]^。而异隐丹参酮在提升LC3Ⅱ表达的同时，降低p-mTOR的活性，作用于AKT/mTOR信号通路提高自噬泡的聚集^[15]^。另一种三萜类化合物——齐墩果酸，可在对正常支气管上皮低毒状态下降低肺癌细胞的活性。并且其衍生物SZC015与隐丹参醌的作用类似，可升高H322细胞中ROS水平，抑制AKT/NF-κB通路^[16]^，上述单体及衍生物均通过诱导自噬性死亡抑制NSCLC。

中药成分中部分生物碱及酸性物质，如甲基莲心碱、哈儿碱、苦参碱以及新藤黄酸也可通过调控PI3K/AKT/mTOR及RAS/RAF/MEK/ERK等经典自噬信号通路中的关键分子，增强NSCLC的自噬活性。甲基莲心碱来源于莲花莲心，该双苄基异喹啉类生物碱在A549细胞中积聚ROS并降低抗氧化剂GSH的表达，抑制PI3K/AKT/mTOR通路并促进LC3-Ⅰ向LC3-Ⅱ的转化，升高了自噬水平并降低了增殖能力^[17]^。哈儿碱（别名骆驼蓬碱）为一种β-咔啉类生物碱，广泛存在于中药苦木中，其药用价值表明它激活A549细胞系的ERK1/2旁路诱发自噬，通过自噬而非凋亡介导癌细胞的死亡^[18]^。苦参碱是“复方苦参碱方”的有效组分，该方于1995年被CFDA批准用于NSCLC以及肝癌的抗癌联合治疗。Wang^[19]^的研究团队证实，多数共同含有苯并-α-吡喃酮结构的苦参碱衍生物均具有一定的抗癌活性，尤其是化合物5i可抑制mTOR信号通路、上调P27并下调CD44和cyclin D1诱发自噬及G_1_期阻滞。抑制自噬可阻断如上生物碱介导的抑癌进程，并且它们在有效剂量范围内毒副作用更小，选择性更高，具备一定的治疗潜力。

在自噬的调节过程中，尚存在新藤黄酸类弱酸性物质，其主要来源于藤黄分泌的干燥树脂。除外经典的导泻、催吐和驱虫等功能，有研究^[20]^也证实新藤黄酸可诱导肺癌细胞系死亡。体外实验中，随其应用时间的延长，逐渐引发NSCLC胞浆内空泡形成，升高Beclin 1及ATG的表达，降低P70S6K磷酸化水平，提示mTOR途径受抑，自噬活性升高。虽然在自噬的晚期进程中，它可抑制溶酶体和自噬体的融合阻断内容物的降解，但新藤黄酸诱发的自噬功能失调从一定程度上介导了癌细胞的死亡。

中药来源的槲皮素、姜黄素等天然膳食多酚也有助于机体对自噬、凋亡、氧化应激和衰老的调节。有文献证实随着金丝桃苷，又称槲皮素-3-O-β-D-半乳糖苷，应用剂量的增大，A549中自噬小体逐渐增多，ERK1/2活性逐渐增强，肺癌细胞的生长受到抑制^[21]^。而姜黄素属于姜黄提取物中多酚科超家族的黄色中药成分，具备抗氧化和抗炎的属性，并通过激活AMPK信号通路激活自噬发挥对肺腺癌的抑制作用^[22]^。衍生物二去甲氧基姜黄素（bisdemethoxycurcumin, BDMC）不仅克服了姜黄素生物利用度较低的限制，而且在诱导凋亡的同时，抑制高转移性大细胞肺癌细胞系（95D细胞）中Hedgehog信号途径，诱发死亡性自噬^[23]^。衍生物HBC（hydrazinobenzoylcurcumin）也可通过促进A549细胞的吞噬泡招募LC3-Ⅱ，降低LC3-Ⅰ/LC3-Ⅱ的比率，介导自噬溶酶体的形成^[24]^。上述活性单体抑制肺癌细胞活性和诱导凋亡的能力可被自噬抑制剂逆转。

作为PI3K/AKT/mTOR通路的抑制剂，重楼皂苷、桔梗皂苷和麦冬皂苷可呈剂量依赖性地诱导NSCLC发生自噬。重楼皂苷（paris saponin Ⅱ, PSⅡ）为中药重楼中提取的甾体类皂苷，可激活ROS非依赖性自噬，抑制NSCLC的增殖。该自噬的发生主要与JNK的激活和mTOR的抑制相关。并且，在接触自噬阻断剂CQ后，可同时降低凋亡水平，提示PSⅡ通过上调JNK/PI3K通路激活自噬并诱导凋亡^[25]^。从桔梗分离得到的三萜类皂苷—桔梗皂苷D（platycodin-D, PD）目前广泛应用于各种呼吸系统疾病的中医诊疗方案，甚至可作为乳腺癌、胃癌、结肠癌等多种癌症的替代方案。除外引发凋亡，PD同样可通过提高H460和A549细胞内JNK及p38 MAPK信号通路的磷酸化水平诱导NSCLC发生自噬从而发挥抑癌作用^[26]^。另外，Xiong等^[27]^先前发现“柯金燕处方”可抑制PI3K/AKT通路，不仅抑制肺癌模型的增殖，也可改善裸鼠的身体状况。随后，在H157和H460细胞系中的结果证实，作为该处方衍生物的的重要活成分之一，麦冬皂苷B正是通过抑制AKT在Ser473、Thr308位点的磷酸化升高LC3-Ⅱ水平，引发NSCLC自噬^[28]^。而且在NSCLC异种移植物中，单独应用的生物学效应和处方一致^[29]^。自噬抑制剂减弱自噬活性的同时可降低凋亡水平，表明三类皂苷类化合物多通过自噬和凋亡发挥协同抑癌的作用。

另外，乙酰化的桔皮素、白花丹素也可在诱导自噬的同时诱导凋亡，但厚朴酚与补骨脂定对凋亡的影响并不显著。桔皮素是中药橘皮中具有抗癌活性的黄酮类化合物，可诱导G_1_期阻滞和凋亡的发生抑制增殖，常通过乙酰化修饰来提高其生物利用度^[30]^。乙酰化的桔皮素活性成分：5-乙酰氧基-6, 7, 8, 4'-四甲氧基黄酮（5-acetyloxy-6, 7, 8, 4'-tetramethoxyflavone, 5-AcTMF）被证实具有抗血管形成、抗炎等多种药理活性^[31]^。来源于白花丹的萘醌类化合物—白花丹素与5-AcTMF具有相似的抗癌活性，均可通过抑制mTOR通路诱导自噬、通过内源性线粒体凋亡通路引发凋亡，实现对NSCLC的抑制^[32]^。但厚朴的提取物厚朴酚对H460细胞生长的抑制作用主要由自噬而非凋亡信号介导，在引发吞噬泡形成的同时抑制DNA合成，潜在信号通路为PI3K/PTEN/AKT通路^[33]^。另外，补骨脂定作为一种具有细胞毒性的补骨脂提取物，可显著提升A549细胞胞内ROS水平、升高LC3-Ⅱ/LC3-Ⅰ的比值，甚至可抑制化学致癌性，但与厚朴酚类似，其对凋亡的影响并不显著，抑制自噬或清除ROS后可降低它的细胞毒性^[34]^。总之，诱导自噬性死亡在部分中药活性成分抑制NSCLC恶性生物学行为中发挥着重要的作用。

### 2.2 多种天然中药活性成分诱发保护性自噬（protective autophagy）促进NSCLC生长

其它萜类、皂苷类、膳食多酚类化合物，例如甘草次酸、甘草查而酮A、薯蓣皂甙、总丹参酮、儿茶酚及β-榄香烯对自噬活性的调控所发挥的生物学效应与上述物质不尽相同。

从中草药甘草中分离得到的活性成分甘草次酸（glycyrrhetinic acid, GA）及甘草查尔酮A（licochalcone A, LCA）也被证实可诱发NSCLC发生保护性自噬。Tang等^[36]^发现GA可激活IRE1α/JNK/c-Jun旁路，通路抑制剂协同自噬抑制剂可强化对NSCLC增殖的抑制，反之亦然^[35]^。其研究团队随后也证实LCA可引起A549和H1299发生自噬。二者共同升高LC3-Ⅱ的表达，促进癌细胞的存活。

同样，薯蓣皂甙、总丹参酮（total tanshinones, TDT）和绿茶提取物（green tea extract, GTE）呈现出与其它皂苷、丹参酮和膳食多酚不同的生物学活性。Hsieh等^[37]^提出，从薯蓣科植物根茎中提取的甾体皂苷——薯蓣皂甙呈剂量依赖性地增强A549及H1299的自噬活性，该趋势与ERK1/2和JNK1/2通路活性增强的趋势相一致。其次，一项针对TDT的研究^[38]^提出，其4种成分可使95D肺癌细胞内ROS累积从而诱发保护性自噬。而GTE诱导的自噬使A549细胞对低、高浓度的GTE表现为低应答状态，也提示自噬或许为一种抵抗毒性的自我保护机制^[39]^。自噬抑制剂逆转自噬可降低NSCLC细胞的活力并提升三者诱导的凋亡比率，促进癌细胞死亡。

与此同时，与其它癌症中的研究一致，中草药植物温莪术挥发油的提取成分β-榄香烯也被证实可在NSCLC中通过抑制mTOR通路，呈剂量依赖性地诱发凋亡，随之诱发强烈的保护性自噬反应^[40]^。因而，对NSCLC患者，联合自噬抑制剂和上述天然活性单体的用药方案很可能以高效低毒的特性成为未来NSCLC特异性治疗的备选方案。

## 3 多种传统中药单体及其衍生物诱发自噬影响NSCLC的药物敏感性

目前，随药物覆盖时间的延长，逐渐产生的抗性降低了NSCLC患者的临床获益^[41]^，使之处于尴尬的境地。研究^[42]^表明靶向治疗或放化疗多可激活自噬，协助癌细胞充分利用周围成分适应环境的改变，成为耐药发生的重要机制之一。但令人欣喜的是，研究指出包括雷公藤红素、免疫调节蛋白GMI、甲基莲心碱、槲皮素、桑白皮、白藜芦醇及异鼠李素在内的多种中药天然活性成分可调控NSCLC耐药株的自噬活性，改变对相关药物的反应性。并且，同自噬在NSCLC中发挥的多样化作用相似，天然活性化合物对其耐药性的调节也因时而异。

### 3.1 天然中药活性成分提升NSCLC用药敏感性

天然活性化合物诱导死亡性自噬或抑制保护性自噬，可增强耐药株的药物反应性，降低耐药的发生。

研究^[43]^证实钙离子介导的信号通路为克服MDR的潜在靶标。雷公藤红素及免疫调节蛋白GMI即可通过胞内钙离子水平调节NSCLC的死亡性自噬活性。利用免疫荧光技术，有研究证实从草本植物雷公藤中提取的雷公藤红素可呈剂量依赖性的诱导吉非替尼耐药的H1650和H1975细胞发生钙离子介导的自噬反应，同时还可降解EGFR^[44]^，减少由EGFR点突变（T790M）的存在导致敏感株产生耐药性的可能性^[45]^。来源于小孢子灵芝的免疫调节蛋白GMI也可通过钙离子介导的信号途径诱导大量的自噬小体在A549细胞中聚集。体内实验^[46]^提示口服GMI可显著抑制裸鼠A549异种移植物的生长，体外肺癌细胞模型^[47]^也证实mTOR通路启动的自噬性细胞死亡的存在。更重要的是，GMI诱发的自噬可抑制铂类化合物耐药的NSCLC细胞株的生长，甚至增强卡铂介导的凋亡^[48]^。相较于化疗药物，雷公藤红素和GMI以更低的副反应诱发高水平的自噬性死亡，提升耐吉非替尼或铂类化合物的NSCLC患者的生存质量。

与此同时，甲基莲心碱、槲皮素、桑白皮提取物、白藜芦醇及其衍生物也被证实可增强化疗药物的疗效。研究者^[49]^以胞内酸性液泡的出现为肺癌细胞自噬性死亡发生的标识，证实甲基莲心碱这一生物碱与阿霉素连用不仅可降低阿霉素的毒副作用，还可在抑制肺癌的同时提升其抗癌潜力。针对槲皮素的研究^[50]^也发现，它可增强人肿瘤坏死因子相关凋亡诱导配体（tumor necrosis factor-related apoptosis-inducing ligand, TRAIL）治疗介导的肺癌细胞凋亡过程，而自噬阻断剂可抑制槲皮素的增效效应，提示自噬可作为槲皮素与TRAIL协同抗癌的潜在机制。桑白皮为桑科植物桑的干燥根皮，其提取物主要包括桑辛素、异戊烯类黄酮、香豆酮等有效成分，可抑制癌细胞的转移，诱发细胞毒性。有研究者^[51]^首先证实随桑白皮水提物暴露时间的延长，A549细胞逐渐产生自噬溶酶体。而且，在其作用6 h后联合使用顺铂，可在降低顺铂使用程度的情况下增强对肺癌细胞的毒性。该情形同样适用于吉西他滨和紫杉醇。因而，预使用桑白皮提取物可诱导肺癌细胞发生自噬，提高顺铂等药物的疗效。

另外，白藜芦醇（resveratrol, Res）及其衍生物除上述增敏功效外，也可通过抑制保护性自噬抑制耐药的发生。具体来说，Res和卡铂联用通过引发A549细胞自噬的发生，不仅增强卡铂的疗效，也可协同诱导凋亡^[52]^。而Res和吉非替尼联用通过上调下游基因，诱导自噬、凋亡和细胞周期阻滞，协同抑制吉非替尼耐药株PC9/G的增殖。且自噬可增强联合用药后胞内吉非替尼的聚集，抑制EGFR的磷酸化^[53]^。有趣的是，Res对保护性自噬的阻滞作用也在mTORC1过度活化的肺癌细胞中被发现。应用雷帕霉素会诱发癌细胞保护性自噬的上调影响疗效。而联合应用Res和雷帕霉素相对单一用药，可恢复对AKT信号通路的抑制，从而有效地阻断mTOR通路过度活化的癌细胞中保护性自噬的发生，促进凋亡，实现抑制肿瘤生长和转移的作用^[54]^。除此之外，白藜芦醇的衍生物也在NSCLC自噬活性的调节中占有一席之地。衍生物3, 4, 4'-THS可抑制A549中mTOR依赖的旁路，虽并不影响自噬通量，但提高LC3-Ⅱ的表达并促进GFP-LC3标记的自噬体形成^[55]^。并且，同样是在吉非替尼耐药的细胞株中，衍生物(Z)3, 4, 5, 4'-trans-tetramethoxystilbene可通过提高钙离子水平、激活AMPK和JNK通路、抑制mTOR通路诱导耐药株发生自噬和Caspase非依赖性凋亡，从而减弱耐药株的活性^[56]^。总之，上述中药天然活性成分与相关药物的联合使用或预处理，为NSCLC提供了一种缩短疗程、降低剂量的用药新思路。

### 3.2 天然中药活性成分提升NSCLC用药耐药性

同时，保护性自噬的激活也与药物抗性的获得密切相关。提高的自噬水平在一定程度上通过降低免疫活性或增加癌细胞在缺氧环境下的生存能力，成为NSCLC的一种耐药机制^[57]^。正如当抑制异鼠李素（isorhamnetin, ISO）引起的保护性自噬时，可增强ISO对肺癌细胞的化学疗效，通过线粒体膜电位改变诱发的凋亡性细胞死亡比例也随之增加，最终抑制增殖及克隆的形成。因此，研究者认为ISO可能通过诱导保护性自噬的发生促进NSCLC产生耐药性^[58]^。

总的来说，中药单体及其衍生物通过提高或降低NSCLC的自噬活性，促进或抑制NSCLC耐药株的化疗增敏，对克服NSCLC的临床耐药具有举足轻重的价值。

## 4 总结和展望

随着对天然中药抗癌活性研究的不断深入，众多中药来源的天然活性化合物在NSCLC治疗中发挥的可塑性角色逐渐得到肯定，甚至联合天然产物和传统药物的组合方案较单一疗法表现出更强的抗癌活性和更低的毒副作用。目前，自噬及自噬性细胞死亡在肺癌的发生发展中发挥的作用得到大量实验学证据的支持。最近的研究也表明，在NSCLC中，自噬可成为众多中药来源的天然产物作用的新靶标，甚至可作为中药调节NSCLC耐药性的驱动机制之一，但具体的机制有待诠释。其中，中药来源的部分萜类化合物、皂苷类化合物、生物碱、酸性物质、天然膳食多酚以及其它天然活性成分多被证实可参与调控如PI3K/AKT/mTORC1、AMPK/mTOR、Bcl-2/Beclin 1、EGFR/Beclin 1和RAS/RAF/MEK1/2/ERK1/2等自噬相关信号通路（图 2）。

**2:**
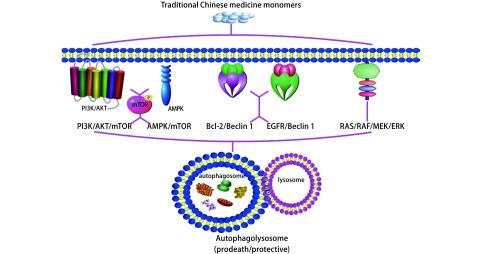
中药单体调控NSCLC自噬相关信号通路 Traditional chinese medicine monomers regulate autophagy signaling pathways in non-small cell lung cancer

引而伸之，中药活性成分是否可通过调节自噬成为克服NSCLC耐药性的突破口，亦或诱发相似的毒副作用，仍需进一步的论证。并且多组分、多靶点、多途径的特点也给中药单体生物学活性的研究蒙上了一层神秘的面纱，如何高效地分离得到具体的效用成分，进而应用于肺癌的临床治疗，仍需克服诸多挑战。但不可否认的是，在探索新型抗癌药物的征程中，中药天然抗癌活性成分联合化疗和靶向治疗的用药方案或许可成为NSCLC患者治疗的新选择。
